# Diagnostic Performance of Biomarkers for Perioperative Hypersensitivity Reactions in Adults: A Systematic Review and Meta-Analysis on Tryptase and Histamine Dosing

**DOI:** 10.3390/diagnostics16071013

**Published:** 2026-03-27

**Authors:** Cristina Petrișor, Cătălin Constantinescu, Robert Szabo, Vlad Dăncilă, Nadia Onițiu-Gherman

**Affiliations:** 1 Anesthesia and Intensive Care II Department, “Iuliu Hațieganu” University of Medicine and Pharmacy, 400012 Cluj-Napoca, Romaniavlad.dancila@elearn.umfcluj.ro (V.D.); 2 Immunology and Allergology Department, “Iuliu Hațieganu” University of Medicine and Pharmacy, 400012 Cluj-Napoca, Romania

**Keywords:** perioperative hypersensitivity, perioperative anaphylaxis, sensitivity, specificity, diagnostic performance, diagnostic accuracy, tryptase, histamine

## Abstract

**Background:** The clinical intra-anesthetic changes of perioperative hypersensitivity (POH) are not specific and require a thorough differential diagnosis with other mimicking conditions. Biomarkers such as tryptase and histamine provide supportive evidence for POH. From the suggested cutoffs, a common decision threshold has not been validated for use in daily practice. The aim of this systematic review and meta-analysis is to identify biomarkers investigated for POH and to evaluate their diagnostic performance. **Methods:** This meta-analysis included original diagnostic accuracy studies comparing patients with clinically suspected POH and controls with no signs of intraoperative hypersensitivity reactions, in whom allergy biomarkers were evaluated, aiming to investigate diagnostic performance of the assays. Data was pooled to evaluate sensitivity and specificity. **Results:** In seven studies on tryptase and three studies on histamine dosing for the diagnosis of POH/POA, different fixed or dynamic thresholds for positivity were proposed. For tryptase, fixed thresholds had 59.8% sensitivity and 95.2% specificity for an optimal cutoff of 12.68 ng/mL, while dynamic thresholds yielded 77.2% sensitivity and 88.5% specificity. For histamine, fixed cutoffs presented 78% sensitivity and 85% specificity, while dynamic thresholds investigated in a single study yielded 78.2% sensitivity and 91.1% specificity. Estimates for histamine are unreliable due to limited data. **Conclusions:** From published data, tryptase is clearly the most robust biomarker, dynamic thresholds boost sensitivity without major specificity loss and confirm the added diagnostic value of relative changes over fixed absolute cutoffs. Preliminary results suggest that histamine might have optimal diagnostic performance, but estimates are severely limited by small sample sizes.

## 1. Introduction

Perioperative hypersensitivity (POH) is a reaction with multiple possible mechanisms and life-threatening potential occurring during the perioperative period, and the most severe cases are referred to as anaphylaxis (POA) [1]. Anaphylaxis is a severe systemic hypersensitivity reaction with sudden-onset airway, breathing and circulatory symptoms [2,3,4]. The incidence of POH/POA is approximately 1 in 10,000 procedures [4,5,6,7,8].

POH requires prompt recognition and treatment [9]. Anaphylaxis is recognized mainly through clinical criteria, which lack specificity [4]. The diagnosis is based on respiratory and cardiovascular compromise, along with possible skin changes like erythema, wheals, pruritus, and edema. The clinical systemic manifestations include, but are not limited to, severe hypotension, arrhythmia, bronchospasm with prolonged expiratory time, gas trapping, wheezing, desaturation, alterations in end-tidal CO_2_, and increases in ventilatory pressures. In the most severe cases, ST segment changes, convulsions and cardiac arrest can appear suddenly [10]. These non-specific changes need to be distinguished from other conditions that mimic intraoperative POH/POA, including the pharmacological effects of drugs such as vasodilation and decreases in blood pressure and tachycardia, as well as mechanical stimulation of the airway during intubation, the effects of anesthetic and surgical procedures or other causes of shock and respiratory insufficiency, drug toxicity or medical errors, or worsening of previous medical conditions. Anesthesia and surgically induced mimics that lead to intraoperative hypotension or bronchospasm need to be differentiated and excluded [1,11,12]. In some reactions in which signs and symptoms are mainly cardiovascular, an immediate hypersensitivity reaction may not be suspected and hypersensitivity might be overlooked or missed [9,10].

POH/POA imply diverse underlying immunological mechanisms which may involve IgE-mediated reactions or reactions based on alternative activation routes like IgG-mediated or mass-related G-protein coupled receptor X2 (MRGPRX2) reactions. Each of these leads to a final common pathway that involves the activation and degranulation of mast cells and basophils, followed by the subsequent release of their stored mediators in the circulation through exocytosis. Biomarkers such as tryptase and histamine provide supportive evidence for POH/POA [13,14]. Serum tryptase is the most analyzed mediator in the acute phase of anaphylaxis [15]. Tryptase was proposed as a possible mediator of anaphylaxis, in the 90s, when a commercial assay was released, with the fluorescent enzyme immunoassay providing results in µg/L or ng/mL for total tryptase [9,16]. To confirm the clinical diagnosis during the acute event, serial blood sampling for tryptase measurements is indicated by current guidelines [4,17]. Still, serial tryptase measurements are seldom implemented and a common decision threshold has not been adopted in daily practice [4]. The transient increase in serum tryptase has been proposed with different decision thresholds, such as 1.35 × basal tryptase (sBT), acute tryptase levels (sAT) > 1.2 × sBT, sAT > 3 + sBT, sAT > 11.4 ng/L, sAT > 14 ng/L, 13.5 µg/L or 8.4 µg/L [4,9,13,15,16,18,19,20].

In 2012, a consensus formula for the diagnosis of mast cell activation syndromes, irrespective of etiology, was proposed and was thereafter validated in clinical practice by several independent groups [21]. This consensus formula stipulates that, to confirm mast cell activation, sAT values should be higher than 1.2 × sBT + 2 [22]. Current data endorse the consensus formula for the detection of mast cell degranulation in adult POH [16]. This remains a recommended gold standard in the evaluation of severe anaphylaxis, being a condition that implies mast cell degranulation. However, there is no validated threshold for POH [23]. Even though the consensus definition has been proposed for mast cell activation syndromes, its validation for POH has been investigated in few studies having different methodologies.

The aim of this systematic review is to identify biomarkers that have been investigated for POH/POA and to evaluate their diagnostic performance in terms of sensitivity and specificity to confirm the POH/POA diagnosis using different cutoffs.

## 2. Materials and Methods

We conducted a comprehensive systematic search of PubMed, Web of Science (WOS) and the Cochrane Library to identify studies in the English language evaluating the diagnostic performance of biomarkers in the investigation of POH/POA, last searched on 22 January 2026 (Supplementary File S1).

Study inclusion criteria:

• Type of studies: original studies.
• Methodology of studies: diagnostic accuracy studies.
• Included populations: adults.
  - patients with suspected intraoperative hypersensitivity or anaphylaxis (POH/POA) as suggested by clinical criteria.
  - controls with no signs of intraoperative hypersensitivity reactions or other conditions but not fulfilling clinical criteria for POH/POA.
• Intervention/exposure: measurement of specific allergy biomarkers, including, but not limited to, acute and baseline serum tryptase, plasma histamine, and others.
• Outcomes: diagnostic performance; accuracy; sensitivity; specificity; positive/negative predictive values, receiver operating curve (ROC) analysis.

Study exclusion criteria:

• Types of studies: reviews, letters, cohorts of cases, editorials, epidemiologic studies.
• Methodology of studies/content: studies not evaluating diagnostic performance or accuracy.

**Figure 1:**
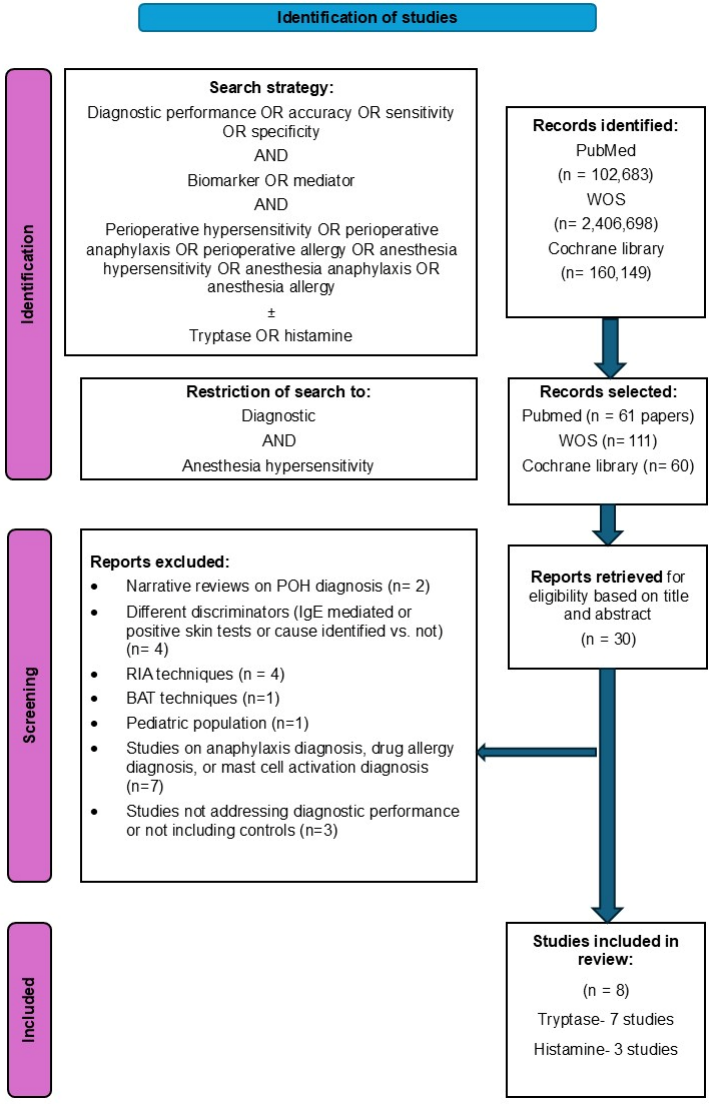
Flow diagram of the study. WOS = Web of Science; POH = perioperative hypersensitivity; RIA = radio-immunoassay; BAT = basophil activation test.

The selection process followed the Preferred Reporting Items for Systematic Reviews and Meta-Analyses (PRISMA) guidelines (Figure 1) [24].

In the first round, a comprehensive search automatically included studies with the following key terms combined:

Diagnostic performance OR Diagnostic accuracy OR Sensitivity OR Specificity

AND

Biomarker OR Mediator

AND

Perioperative hypersensitivity OR Perioperative anaphylaxis OR Perioperative allergy OR Anaesthesia hypersensitivity OR Anaesthesia anaphylaxis OR Anaesthesia allergy

±

Tryptase OR Histamine

Among the identified papers, the search was restricted based on “Diagnostic performance biomarker/mediator AND Perioperative hypersensitivity OR Anaphylaxis OR Allergy” criteria and papers were selected to be screened further.

In the second round, the authors independently screened the retrieved papers based on titles and abstracts, and only 30 studies discussing diagnostic performance or sensitivity or specificity of biomarkers for the diagnostic performance of POH/POA were retained.

In the third round, the papers were evaluated in terms of their compliance with the aim of this systematic review and meta-analysis and we excluded papers on:

• Diagnostic performance of biomarkers to discriminate between IgE-mediated reactions and non-IgE mediated reactions or between patients with positive skin tests versus negative skin tests. We excluded four papers, since both IgE- and non-IgE-mediated reactions, or reactions in patients confirmed by positive skin tests or not, can present mast cell degranulation [8,10,25,26].
• Methods using radio-immunoassay (RIA) techniques have been in place in the past but are no longer available in practice. We excluded four papers using RIA techniques and a paper using a complex in vitro technique for the diagnosis of succinylated gelatine hypersensitivity, as the assay is not widely available [27].
• One study evaluating the diagnostic performance of tryptase in pediatric POH/POA was excluded because it demonstrated significant differences compared to adults [28].
• Studies on the diagnostic performance of biomarkers for hypersensitivity or anaphylaxis in general that did not focus on the perioperative period but rather on in-hospital events. Also, we excluded studies in which multiple etiologies were included, among which were small samples of POH/POA [9,14,15,19,21,29,30].
• We excluded a study on different biomarkers, in which values were demonstrated to discriminate patients with POH/POA versus controls, but no sensitivity and specificity values were calculated [31], and two epidemiologic studies in which controls were not included [11,32], thus diagnostic performance was not evaluated.
• Narrative reviews on POH diagnosis were excluded as well [1,20].

Diagnostic performance (accuracy, sensitivity, specificity) of biomarkers for the diagnosis of POH/POA, in which the discriminator was presence versus absence of disease (patients with POH/POA versus controls with no POH/POA), was evaluated in a total of eight studies that were included in this meta-analysis, including seven tryptase and three histamine diagnostic accuracy studies, aiming to evaluate the pooled sensitivity and specificity of these two biomarkers for POH/POA diagnosis.

For data extraction and quality assessment, two-by-two contingency tables (TP = true positive, FP = false positive, FN = false negative, TN = true negative) were extracted for each biomarker, cutoff, and study. Risk of bias was assessed using QUADAS-2 domains (patient selection, index test, reference standard, flow and timing), with most studies rated as low risk (retrospective case–control designs were predominant) (Supplementary File S2).

Statistical analyses were performed in R (2024.04.1+748) using packages diagmeta and mada. Fixed cutoffs (numeric thresholds) were pooled using diagmeta with the common slopes (CS) model to accommodate small data and multiple cutoffs per study. Dynamic thresholds (non-numeric) and overall data (aggregated per study to avoid double-counting) were pooled using bivariate random-effects models (reitsma) with continuity correction of 0.5 for zero cells. Pooled sensitivity, specificity, false positive rate, and area under the summary ROC curve (AUC) were calculated with 95% confidence intervals. Heterogeneity was quantified using Zhou and Dendukuri and Holling approaches. All analyses were stratified by biomarker and threshold type (fixed or dynamic).

## 3. Results

From the eight studies addressing biomarkers’ sensitivity and specificity, seven studies evaluated tryptase and three studies evaluated histamine dosing performance for the diagnosis of POH/POA, using different fixed or dynamic thresholds for positivity throughout the studies (Table 1). In two of the studies, both tryptase and histamine dosing were performed.

**Figure 2:**
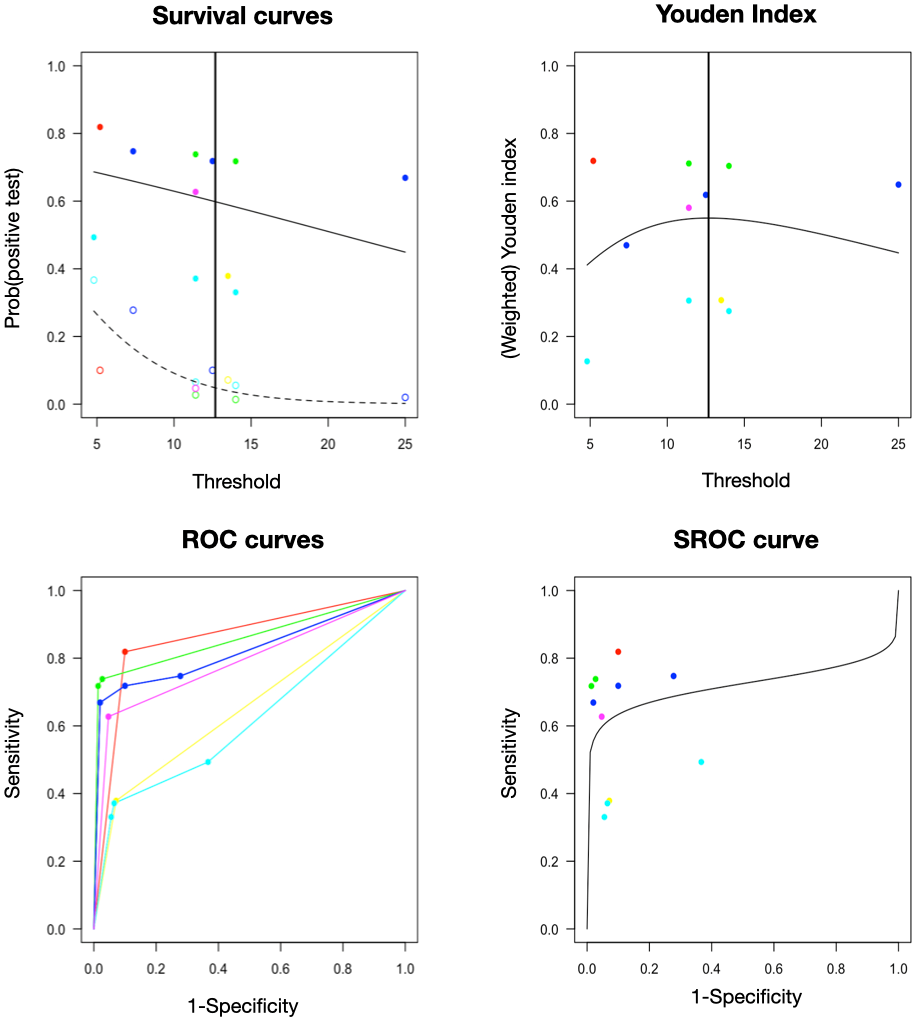
Diagnostic performance of serum tryptase using fixed cutoffs in perioperative anaphylaxis (6 studies, 11 cutoffs). (**Top left**) Survival curves showing the probability of a positive test (elevated tryptase) versus threshold, clinically relevant for understanding how stricter cutoffs reduce false positives but may miss milder cases. (**Top right**) Weighted Youden index (sensitivity + specificity − 1, a measure of overall diagnostic accuracy balancing test performance) versus threshold (peak near 12.7 ng/mL). (**Bottom left**) Individual study ROC curves. (**Bottom right**) Summary ROC curve with pooled estimate (Sens 0.60, Spec 0.95 at optimal cutoff 12.68 ng/mL; AUC ≈ 0.72). The optimal cutoff was derived as the value maximizing the weighted Youden index in the diagmeta multiple-threshold model.

For tryptase, six studies evaluated different fixed thresholds, with 59.8% (42.7–74.8%) sensitivity and 95.2% (81.3–98.9%) specificity for an optimal cutoff of 12.68 ng/mL (Figure 2). The AUC of approximately 0.72 indicates fair–good accuracy. The tight ellipse and the focus on low false positive rates confirm high pooled specificity (0.95) with moderate sensitivity (0.60) at the optimal cutoff. The curve’s shape supports tryptase as a strong rule-in test (high specificity) but it is not ideal for rule-out tests (lower sensitivity).

**Figure 3:**
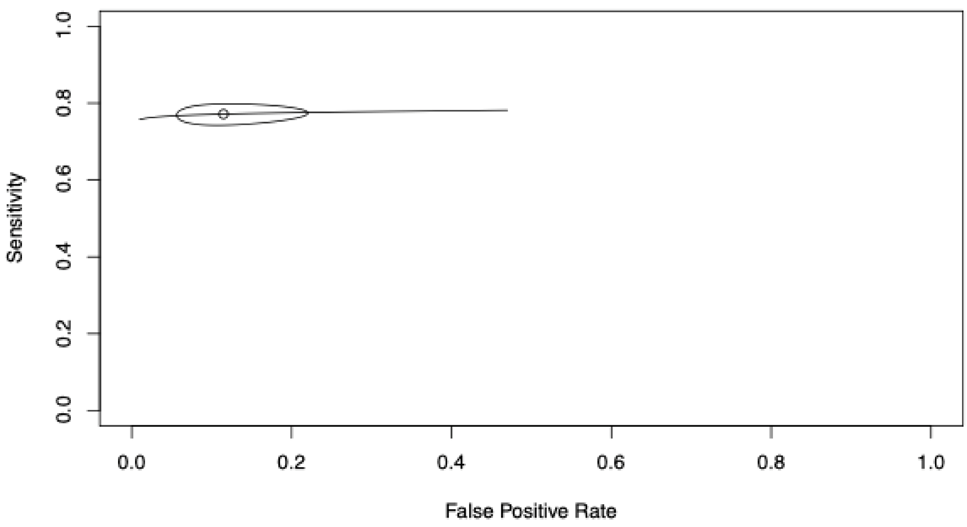
Summary Receiver Operating Characteristic (SROC) curve for tryptase using dynamic thresholds in perioperative anaphylaxis (5 studies, 12 entries). The summary point indicates pooled sensitivity 77.2% (74.9–79.3%) and false positive rate 11.5% (6.5–19.5%), with AUC 0.774. The confidence ellipse represents uncertainty in the pooled estimate, incorporating interstudy heterogeneity (I^2^ 0–43.8%); a narrower ellipse suggests relatively consistent performance across studies despite varying dynamic definitions.

A total of five studies evaluated tryptase dynamic thresholds, with 77.2% (74.9–79.3%) pooled sensitivity, 88.5% (80.5–93.5%) specificity and AUC 0.774 (Figure 3). The narrow confidence ellipse reflects low-to-moderate heterogeneity (Figure 3). The curve hugs the top-left corner very well in the clinically relevant low false positive rates zone (FPR < 0.2), then plateaus at sensitivity ~0.77–0.78 before dropping more steeply at higher false positive rates. This indicates excellent rule-out performance (high specificity) with good rule-in performance (sensitivity ~77%).

The consensus formula sAT > 1.2 × sBT + 2 was evaluated in all five studies on tryptase dosing using dynamic thresholds, demonstrating sensitivity 75–78.6% and specificity 86–100% (Table 1). Each study evaluated the performance of the consensus formula compared to other thresholds that varied widely among studies. The consensus formula represented the most frequently used criteria for positivity among the dynamic thresholds.

**Figure 4:**
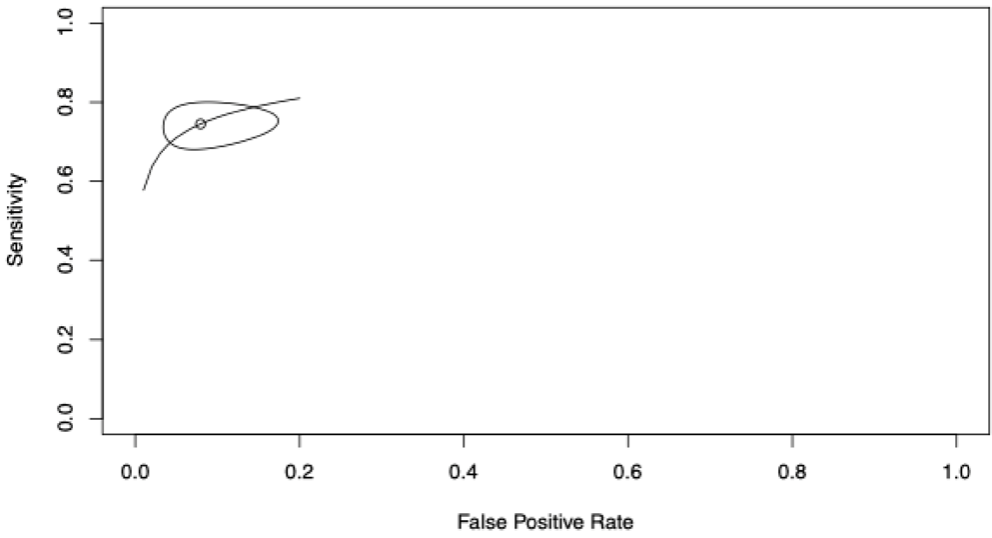
Summary Receiver Operating Characteristic curve for overall tryptase performance (fixed + dynamic thresholds combined and aggregated per study; 7 studies). The summary point indicates pooled sensitivity 74.5% (69.4–79%), with AUC 0.852. The confidence ellipse represents uncertainty in the pooled estimate, incorporating interstudy heterogeneity (I^2^ 22.4–78.9%).

Overall pooled sensitivity was 74.5% (69.4–79%) and specificity was 92.1% (85–96%) for tryptase dosing to confirm POH/POA (Figure 4). The summary point (open circle) indicates an AUC of 0.852. The narrow confidence ellipse reflects moderate heterogeneity but reliable pooling in perioperative anaphylaxis. The curve stays well above the diagonal line of no discrimination, with the strongest performance in the low false positive rates zone (false positive rate < 0.2), where specificity exceeds 90%. This shape visually confirms strong overall diagnostic accuracy, with excellent specificity and good sensitivity, the best balance among the three tryptase analyses.

**Figure 5:**
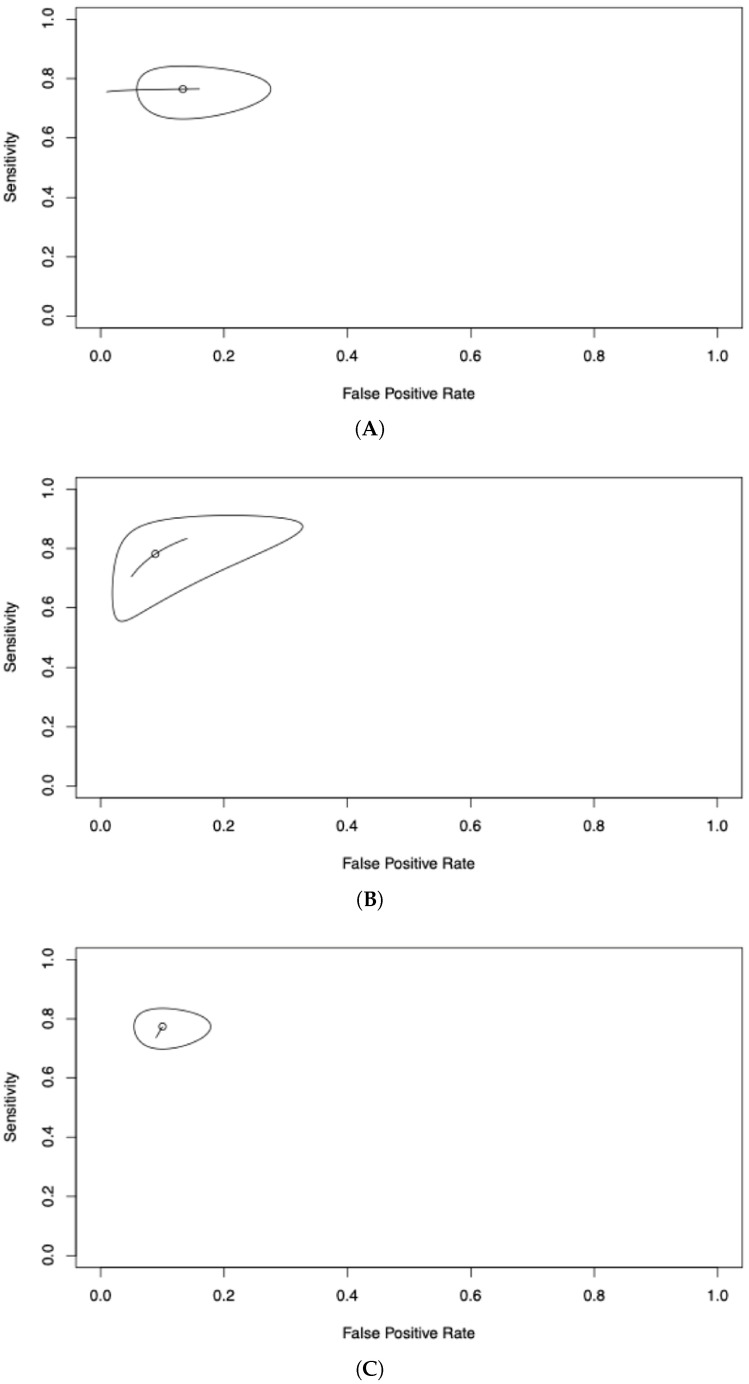
Summary Receiver Operating Characteristic (SROC) curve for histamine. Diagnostic performance for histamine using fixed cutoffs (pooled sensitivity ~78%; specificity ~85%; AUC ~0.80) (**A**); dynamic thresholds (pooled sensitivity 78.2%; specificity 91.1%; AUC of 0.905) (**B**); and overall histamine measurements for POH/POA diagnosis (high apparent AUC = 0.922; zero/low I^2^) (**C**).

For histamine, fixed cutoffs were investigated in three studies, with 78% sensitivity and 85% specificity, both displaying wide confidence intervals (Figure 5A), while dynamic thresholds investigated in a single study yielded 78.2% (60.5–89.3%) sensitivity and 91.1% (73.9–97.4%) specificity (Figure 5B). Overall sensitivity for histamine dosing was 77.4% (71.4–82.4%) and specificity 90% (84–93.9%) (Figure 5C). Estimates for histamine are approximate and unreliable. Results are highly uncertain, as demonstrated by the narrow ellipse in the reitsma plots reflecting precision from small samples, rather than generalizability, due to very small sample sizes and very few primary studies included. Thus, results should be interpreted cautiously.

From the available published data, tryptase is clearly the most robust biomarker, with an overall sensitivity of 0.745 (0.694–0.79) and specificity of 0.921 (0.85–0.96) (AUC 0.852), dynamic thresholds boosting sensitivity without major specificity loss (Table 2). Histamine might demonstrate optimal performance, but all estimates are severely limited by small sample sizes and the low number of diagnostic accuracy studies published so far for POH/POA diagnosis.

## 4. Discussion

Accurate diagnosis of anaphylaxis is essential and starts from a suggestive clinical picture [33]. Distinguishing anaphylaxis is challenging because hallmark symptoms (hypotension, tachycardia, bronchospasm) often overlap with the physiological effects of anesthesia, surgical stress, or pre-existing health conditions [8]. Meeting clinical criteria does not necessarily confirm a diagnosis of hypersensitivity [6]. A thorough and rigorous differential diagnosis is required. Currently, there is interest in exploring biomarkers of anaphylaxis as the release of mediators in the circulation during the acute event might confirm that a true hypersensitivity reaction was responsible for the acute intraoperative event [19].

Quantitative dosing methods for specific mediators are valuable to suggest the hypersensitivity mechanism by demonstrating mast cell degranulation retrospectively. This confirmation might be relevant for further testing. Most studies on POH/POA investigated tryptase and histamine as possible biomarkers. Tryptase and histamine testing has the potential to support or confirm the diagnosis, preventing underdiagnosis [8,14].

Mast cells produce proinflammatory mediators and cytokines and contain cytoplasmic granules that store histamine, tryptase, heparin, prostaglandin, leukotrienes, platelet-activating factor (PAF), cytokines, chemokines and other proteases, all of which are released upon activation, irrespective of different endophenotypic categories like IgE- or IgG-mediated mechanisms, cytokine release, MRGPRX2 or cyclooxygenase-1 (COX-1) inhibition [8,9,14,15,19,21]. Clinical manifestations of hypersensitivity are driven by the release of inflammatory mediators rather than the specific underlying mechanism or triggering agent. Because these mediators represent a common final pathway of degranulation, the resulting clinical picture is indistinguishable across different etiologies.

Detecting mediators of POH/POA helps to confirm that acute intraoperative respiratory and cardiovascular deterioration is indeed caused by a hypersensitivity reaction, even though no single biomarker currently provides an unambiguous diagnosis of anaphylaxis. An ideal diagnostic test must be both sensitive and specific for hypersensitivity reactions, utilize a viable laboratory assay, and show minimal diurnal or interindividual variability [19]. Of the several biomarkers proposed to meet these criteria, tryptase appears to offer the highest diagnostic accuracy [6].

Serum tryptase, the principal mast cell biomarker available, has proven to be valuable in confirming degranulation of mast cells during anesthesia-related immediate hypersensitivity reactions [1,14,20]. For POH/POA, serum tryptase is the most studied biomarker, but is far from ideal [19]. Baseline tryptase is constitutively secreted, representing sBT. The mature forms are actively secreted from the mast cells during degranulation, the tryptase levels during acute episodes of degranulation representing the sum sBT + sAT [4,12,14,20,21]. A laboratory assay may help harmonize diagnosis as the measurement of total tryptases is a unique, simple, cost-effective and reliable analysis [9,11]. Tryptase has a peak level 15–120 min after degranulation, thus measurement should be in this time frame, and values decrease 6–8 h after the acute event [9,10]. All tryptase forms increase vascular permeability, contributing to the drop in blood pressure [1,12].

Fixed thresholds like 11.4 or 14 ng/mL have initially been proposed by the manufacturer for test positivity (Phadia ThermoFisher, Uppsala, Sweden), while other researchers proposed other fixed thresholds like 3, 5, 12, 13.5 or 25 ng/mL [18]. The cutoff point that achieves optimal sensitivity and specificity determines the biomarker’s differentiating ability to discriminate between POH/POA and other intraoperative events [8]. The diagnostic performance is strongly correlated to the threshold value. sBT values are relatively consistent in the life of individuals but present wider interindividual variability. Single tryptase measurements are less sensitive [33]. In addition, the sensitivity of the assay is related to the cutoff and to severity [14]. An apparently low level of sAT does not exclude mast cell degranulation, especially in reactions without a significant drop in blood pressure [16]. In a study including 3400 cases, predefined thresholds like 11.4 or 14 µg/L confirmed the reaction in 41–45.6% of cases, with the authors concluding that these thresholds are insensitive and many patients with anaphylaxis and IgE-mediated reactions having peak tryptase levels within this reference range [11]. A single sAT value does not accurately diagnose POH and paired samples are required to allow interpretation [16].

Due to these shortcomings, it has been suggested that serial measurements might be more useful, reflecting dynamics of serum tryptase. In 2010, the consensus formula (1.2 × sBT + 2) has been proposed for mast cell degranulation, irrespective of the underlying cause [22]. Measuring dynamic values improves the diagnostic of mast cell degranulation [1,4,9,22,35]. Paired acute and baseline serum or plasma tryptase determination has been adopted as the optimal approach in the diagnosis and management guidelines of POH/POA, but a specific consensus cutoff for tryptase is lacking in this setting [1,8,17]. Whether general consensus thresholds apply to POH/POA has been evaluated in few studies. There is room for improvement in the decision threshold for POH [16]. Moreover, the optimum number of tryptase measurements and the best interpretative strategies have not yet been established in perioperative anaphylaxis [11]. The way the increase in tryptase should lead to the diagnosis of mast cell activation varied [18]. Dynamic threshold studies considered the absolute increase in tryptase values (Δ tryptase = sAT − sBT), percentage of increase (Δ tryptase% or tryptase increase = sAT − sBT/sBT), or the consensus formula as possible useful thresholds for POH/POA diagnosis, using different values for these formulas derived from paired sampling of tryptase. Dynamic tryptase evaluation improves detection of tryptase release and should be adopted [32].

This meta-analysis on perioperative anaphylaxis diagnosis in adults demonstrates that serum tryptase remains a valuable diagnostic biomarker, with overall pooled sensitivity of 74.5%, specificity of 92.1% and an AUC of 0.852. Dynamic thresholds yielded the highest sensitivity (77.2%) while maintaining strong specificity (88.5%), confirming the added diagnostic value of relative changes over fixed absolute cutoffs (sensitivity 59.8%, specificity 95.2%). Dynamic thresholds seem to be more accurate than fixed values, which are more prone to misdiagnosing patients, especially in the low reference range. An increase of 3–3.2 µg/L yielded similar results compared to the consensus formula and has good accuracies [11,16]. The interindividual variability in tryptase concentrations might be one of the limiting factors in the use of fixed diagnostic thresholds. From the meta-analysis data, fixed absolute cutoffs sacrifice sensitivity for very high specificity, while dynamic thresholds improve confirmation of positive cases without much specificity loss. Tryptase’s diagnostic potential is maximized with dynamic strategies, that increase sensitivity, which is important to avoid false negative results in this life-threatening disease. The high specificity and moderate sensitivity are typical for tryptase in anaphylaxis diagnosis, having few false positives but missing a proportion of true cases due to lower sensitivity. The high positive predictive value highlights that tryptase dosing is reliable for diagnostic positivity. However, a negative result does not rule out a hypersensitivity reaction. Sensitivity remains a limiting factor for tryptase dosing, and not all reactions are confirmed retrospectively.

Measuring serum tryptase levels is valuable in the diagnosis of anaphylaxis but is unable to detect all anaphylactic reactions [19]. Tryptase could be normal in less severe reactions or when blood samples are extracted too early, so minimal changes do not exclude anaphylaxis [10,25]. The consensus formula is particularly useful for patients with POH and tryptase in the lower range [16]. Two studies on real-world practice confirmed the value of paired tryptase measurements in patients with POA/POH, demonstrating the utility of dynamic measurements in large sample sizes and real-life practice [11,32]. These studies, even though lacking controls, demonstrate that the results of the diagnostic accuracy studies included in this meta-analysis are relevant for anesthesia practice in case of POH/POA events.

Alongside tryptase, histamine acts as a primary mediator of anaphylactic symptoms. However, its utility is limited by its transient half-life: levels peak between 5 and 15 min and normalize after 30 min, necessitating immediate blood sampling [10]. While measuring tryptase is the standard recommendation, Haraguchi et al. argue that there is insufficient evidence to justify preferring tryptase to the exclusion of histamine [33]. Histamine metabolites such as urinary methyl-histamine can also be useful [11].

Histamine has not been studied as much as tryptase and only three studies investigated histamine diagnostic performance in POH/POA [6,33,34]. In this meta-analysis, histamine showed numerically comparable sensitivity of approximately 77–78%, with high specificity of 85–91%, but all estimates are severely limited by small sample sizes and should be regarded as preliminary. Thus, current evidence is insufficient for clinical recommendations regarding histamine. Our findings reinforce the need for larger validation studies. Histamine has potential as a diagnostic biomarker but is constrained by practical challenges (timing, stability) and insufficient validation so far in the perioperative setting.

For both tryptase and histamine, the diagnostic performances of biomarkers for POH/POA are higher compared to their diagnostic performances for other hypersensitivity reactions like environmental agent hypersensitivity, anaphylaxis due to other drugs or food-induced anaphylaxis [13]. This might occur because POH/POA reactions occur under strict medical surveillance and continuous monitoring, thus, diagnostic certainty and optimal timing of sampling are superior compared to hypersensitivity reactions induced by other allergens under no medical supervision.

Several other biomarkers have been analyzed as being linked to anaphylaxis, but their optimal application to POH/POA diagnosis has not been established in the literature [13,29,31]. Serum CD203c+ extracellular vesicles and protein expression level of CD63 and CD203c seem to be useful to confirm succinylated gelatine hypersensitivity [27]. Even though there is preliminary evidence that the measurement of other mast cell mediators may also become important, there is a need to explore other biomarkers and there is a need to test these in the context of POH [19,25].

The strength of our paper comes from the strict focus on POH/POA diagnosis confirmation using biomarkers currently available and investigated in diagnostic accuracy studies. We evaluated the diagnostic performance of biomarkers in terms of sensitivity and specificity in this meta-analysis including studies on the diagnostic accuracy of tryptase and histamine. Other biomarkers have not been evaluated in rigorous studies on POH/POA. We have restricted the search to studies that included well-characterized patients with POA/POH based on clinical criteria and controls with no POH/POA (patients with uneventful anesthesia or patients with other intraoperative events not fulfilling POH/POA criteria).

We have excluded studies in which biomarkers were used to discriminate between patients with IgE-mediated reactions versus those without or patients in whom diagnosis was later confirmed by positive skin tests versus those with negative tests. In these studies, the discriminative value was used for intraclass differential diagnosis and prediction of further tests, performed at 4–8 weeks during the allergology work-up aimed to identify culprits. Tryptase is released during the final common pathway of hypersensitivity and the release is not specific to IgE-mediated reactions. However, tryptase levels seem to be correlated to some extent and are predictive for subsequent positive IgE dosing results or positive skin tests, while also being correlated with severity [10,11,32]. Still, other underlying mechanisms can be responsible for hypersensitivity reactions, and we considered that, by including these studies, we might introduce diagnostic bias. Tryptase and other biomarker values do not discriminate between IgE- versus non-IgE-mediated reactions [28]. Thus, we restricted analysis to papers in which a clear delimitation was done between patients with clinical signs of POH/POA and without. We also excluded papers which included some patients with POH/POA but in larger cohorts with multiple causes of hypersensitivity and the statistical analysis was not targeted on intraoperative events but included in-hospital hypersensitivity reactions or environmental allergies [9,14,29,30]. As has been shown in a single study on pediatric patients with POH/POA, tryptase levels and cutoffs used in the adult population might not be appropriate for children, so we excluded the study on POH in the pediatric population [28].

This meta-analysis evaluates the discriminative diagnostic accuracy of biomarkers for patients with clinically suspected POH/POA versus patients with no clinical signs and symptoms of POH/POA, including well-defined groups in the meta-analysis.

However, the limitations of our review and meta-analysis emerge from the potential selection bias, variability in diagnostic accuracy due to different thresholds of positivity, inconsistent sampling time and retrospective nature of most studies.

One of the limitations of our study is the variability in POH/POA diagnosis and criteria for study enrolment. Since we included studies in a large time frame in which diagnostic guidelines were refined, some authors considered different criteria for clinical symptoms and signs, while others used hypersensitivity clinical scoring systems to increase the certainty of patients’ inclusion. It is currently difficult to remove mimics of anaphylaxis from studies [11]. Diagnostic certainty is enhanced by the hypersensitivity clinical scoring system, which assesses the likelihood of anaphylaxis rather than severity alone [33]. However, variability in severity inclusion criteria complicates cross-study comparisons. By limiting cohorts to grade 3 and 4 reactions, some investigators potentially overlooked genuine cases of mild anaphylaxis [11,12]. Laroche et al. demonstrated this risk, noting elevated histamine in 40% of patients with minor reactions who would otherwise be excluded [12]. Given that biomarker sensitivity drops in less severe events [4], prospective studies must employ subgroup analyses to accurately evaluate performance in mild-to-moderate reactions, which remain in a grey zone [8].

Control group variability is also an important factor to be considered, comprising healthy controls with no anesthesia, patients with no intraoperative events, or patients with other intraoperative events that do not meet POH/POA criteria, like shock due to other causes. Resuscitation maneuvers did not modify mediator concentrations, and histamine and tryptase can differentiate between POH and other forms of shock, which is important for practice [12].

Despite significant heterogeneity in inclusion criteria for patients and controls, the diagnostic accuracy of tryptase measurements remained consistent across multiple centers. This cross-study concordance reinforces the validity and representativeness of the findings.

Another limitation of the included studies is the fact that patients were not screened for alpha-tryptasemia and mastocytosis. Also, we could not retrieve data on non-allergic disorders like renal insufficiency, hematological malignancies, acute coronary syndromes, and inflammatory bowel disease, which are associated with increased tryptase levels. These would influence fixed values of tryptase.

The included studies spanned two decades, in which practices varied, including criteria for diagnosis. Improvement of POH management includes sample storage for deferred analysis, referral for allergy evaluation and collaboration between anesthesiologists and allergists. There might be a wide variation in practice [11]. Still, the clinical picture of POH/POA is constant in time, and the fact that included patients were referred by the attending anesthesiologist makes the results reflect real-life practice. Currently, paired sAT and sBT determination in the setting of suspected POH is not optimally implemented in many hospitals [1]. Sampling time can vary depending on the urgency of the clinical situation, sometimes too late for optimal diagnosis [32]. Since the included studies are predominantly retrospective, the timing of blood sampling might have varied substantially, and, especially for histamine, that has a short half-time, delayed sampling could lead to false negative results.

The role of tryptase in systemic anaphylaxis has been recognized for decades [36]. Though the optimal thresholds for positivity for POH/POA diagnosis varied widely and diagnostic accuracy studies were heterogenous, its use has been recommended by different guidelines throughout the years [37,38,39,40,41], since any additional tools that increase allergological survey sensitivity might help avoid future patient management. The limited number of patients limits the statistical power of individual studies and hampers the identification of all differences and effects [8]. However, in the meta-analysis we have included 601 patients with clinical POH/POA and 206 patients with no clinical signs of POH/POA, which represents an adequate pooled sample for a low-prevalence disease. Unfortunately, evidence is limited due to a lack of well-controlled studies in larger numbers of well-phenotyped patients [14]. High or unclear risk in the flow and timing domain across several studies (retrospective timing inconsistencies) could lead to overestimation of specificity, particularly in perioperative settings where acute sampling is critical. Further studies are needed that include patients and controls to confirm sensitivity and specificity. As individual biomarkers may lack sufficient diagnostic accuracy, a combination of biomarkers may enhance sensitivity and specificity of anaphylaxis diagnosis [13]. Identification of potential new biomarkers can emerge from transcriptomics, metabolomics and proteomics [15].

## 5. Conclusions

From the available published data on POH/POA diagnosis, tryptase is clearly the most robust biomarker, with dynamic thresholds boosting sensitivity without major specificity loss and confirming the added diagnostic value of relative changes over fixed absolute cutoffs. Preliminary results suggest that histamine might have optimal diagnostic performance, but estimates are severely limited by small sample sizes and there is currently no strong superiority over tryptase. Our findings reinforce the need for larger validation studies.

## Figures and Tables

**Table 1:** Studies evaluating the diagnostic performance of tryptase and histamine for the diagnosis of POH/POA.

No.	Reference	Patients	Controls	Threshold	Se	Spe	PPV	NPV	AUC
**Tryptase**									
1	Dybendal, 2003 [3]	18 pts with suspected POH/POA	20 controls	sAT > 13.5 ng/L	66.6	100	100	76.9	
2	Laroche, 2014 [12]	75 pts with POH/POA grades 3 and 4	25 shocks due to other causes	sAT > 7.35 ng/L	92	92	99.4	44.3	
				sAT > 12.5 ng/L	82.7	96	99.7	27.9	
				sAT > 25 ng/L	68	100			
3	Baretto, 2017 [23]	60 pts with clinical criteria	11 pts with no clinical criteria for POH	sAT > 1.2 × sBT + 2	78	91	98	44	-
				% Δ tryptase increase 48.57% × sBT	86.6	90.9	98.1	55.6	0.79
				sAT > 5.2 ng/L	81.3	63.6	92.3	38.9	0.92
4	Vitte, 2019 [4]	64 pts with clinical criteria	21 controls	sAT > 1.2 × sBT + 2	75	86	94	53	
				>1.35 × sBT	81	52	84	48	
				>sBT + 3	72	86	94	50	
				>11.4 ng/L	53	95	97	40	
5	Ebo, 2021 [16]	296 pts with clinically suspected POH/POA	75 pts with uneventful general anesthesia	sAT> 1.2 × sBT + 2	78	95	98	52	0.86
				Δ tryptase (sAT − sBT) > 3.2 µg/L	78	99	99.6	54	0.89
				sAT > 14 µg/L	64	99	99	41	0.81
				sAT > 11.4 µg/L	70	97	99	45	0.84
				% Δ tryptase increase 85% × sBT	75	91	97	48	0.83
6	Takazawa, 2021 [6]	43 pts grade 2 or higher, composite clinical score	12 pts with other intraoperative events	sAT > 1.2 × sBT + 2	78.6	100	100	57.1	
7	Haraguchi, 2024 [33]	45 pts with clinical grade 2 or higher, composite clinical score	30 pts with uneventful anesthesia 12 pts with no POH/POA	sAT > 11.4 ng/L	53	98	96	76	-
				sAT > 14 ng/L	44	98	95	66	-
				sAT > 1.2 × sBT + 2	71	95	94	76	-
				% Δ tryptase increase 52% × sBT	82	93	93	83	0.92
				sAT > 2 × sBT	73	95	94	77	-
				sAT > 4.8 ng/L	80	88	88	80	0.87
**Histamine**									
1	Takazawa, 2021 [6]	43 pts grade 2 or higher, composite clinical score	12 pts with other intraoperative events	Value at 30 min	76.7	100	100	54.5	0.86
				Value at 2 h	79.1	83.3	94.4	52.6	0.86
2	Haraguchi, 2024 [33]	45 pts with clinical grade 2 or higher, composite clinical score	30 pts with uneventful anesthesia 12 pts with no POH/POA	% Δ histamine peak 51%	84	86	86	84	-
				Δ histamine max 0.76	71	95	94	76	-
				Histamine max 1.5 ng/mL	76	88	87	77	-
3	Horiuchi, 2023 [34]	43 pts with clinical grade 2 or higher, composite score	30 pts with uneventful anesthesia	30 min: 1.5 ng/mL	77	100	100	75	0.83
				2 h: 1.1 ng/mL	67	87	87.9	65	0.79

Notes: pts = patients; sAT = serum acute tryptase; sBT = serum baseline tryptase; Se = sensitivity; Spe = specificity; PPV = positive predictive value; NPV = negative predictive value; AUC = area under the curve for ROC analysis.

**Table 2:** Pooled sensitivity and specificity for fixed, dynamic and overall tryptase and histamine dosing for POH/POA diagnosis.

Biomarker/Subset	No. of Studies/ Entries	Pooled Sensitivity (95% CI)	Pooled Specificity (95% CI)	AUC (ROC)	Heterogeneity (I^2^ Range)	Notes
Tryptase— Fixed cutoffs	6 studies/ 11 cutoffs	0.598 (0.427–0.748)	0.952 (0.813–0.989)	~0.72	Not reported (CS model)	Optimal cutoff: 12.68 ng/mL
Tryptase— Dynamic thresholds	5 studies/ 12 entries	0.772 (0.749–0.793)	0.885 (0.805–0.935)	0.774	0–43.8%	Baseline-adjusted strategies
Tryptase— Overall (combined)	7 studies/ 23 entries (aggregated)	0.745 (0.694–0.790)	0.921 (0.850–0.960)	0.852	22.4–78.9%	Best overall balance
Histamine— Fixed cutoffs	3 studies/ 5 entries	~0.78 (wide CI)	~0.85 (wide CI)	~0.80	Not estimable	Very limited data
Histamine—Dynamic thresholds	1 study/ 2 entries	0.782 (0.605–0.893)	0.911 (0.739–0.974)	0.905	0% (1 study)	Extremely limited (unreliable)
Histamine— Overall (combined)	3 studies/ 7 entries (aggregated)	0.774 (0.714–0.824)	0.900 (0.840–0.939)	0.922	0–28.6%	Preliminary; only 3 studies

Notes: AUC values are from ROC curves. Confidence intervals (CIs) for specificity derived from false positive rates; CIs were not directly reported. All analyses used continuity correction (0.5) in reitsma models.

## Data Availability

No new data were created or analyzed in this study.

## Supplementary Materials

The following supporting information can be downloaded at: https://www.mdpi.com/article/10.3390/diagnostics16071013/s1, Supplementary File S1: Table S1. Search strategy from PubMed. Nbr = Number; Table S2. Search strategy from Web of Science; Table S3. Search strategy from Cochrane; Supplementary File S2: Figure S1. QUADAS evaluation for histamine–plot. Figure S2. QUADAS evaluation for histamine- risk of bias domains. Figure S3. QUADAS evaluation for tryptase–plot. Figure S4. QUADAS evaluation for tryptase-risk of bias domains. Supplementary File S3: PRISMA 2020 Checklist.

## Abbreviations

The following abbreviations are used in this manuscript:

POH	Perioperative hypersensitivity
POA	Perioperative anaphylaxis
sBT	Basal tryptase
sAT	Serum acute tryptase
ROC	Receiver operating curve
AUC	Area under the curve
PAF	Platelet-activating factor
PGD2	Prostaglandin D2
MRGPRX2	Mass-related G-protein coupled receptor X2
RIA	Radio-immunoassay
BAT	Basophil activation test
Se	Sensitivity
Spe	Specificity
PPV	Positive predictive value
NPV	Negative predictive value
FPR	False positive rate
CI	95% confidence interval
