# Supplementary material for: Diagnostic Performance of Biomarkers for Perioperative Hypersensitivity Reactions in Adults: A Systematic Review and Meta-Analysis on Tryptase and Histamine Dosing
Source: Diagnostics (Basel). 2026 Mar 27;16(7):1013. doi: 10.3390/diagnostics16071013 (PMC13073476; doi:10.3390/diagnostics16071013)
Supplement: Supplementary file 1 [file diagnostics-16-01013-s001.zip › Supplementary File S1.pdf]

Supplementary file S1

PubMed – last check 13.03. 2026

Table S1.

Ordinal Number	Search Strategy	Nbr. of entries
#1	((((((("diagnosis"[MeSH Terms] OR "diagnosis"[All Fields] OR "diagnostic"[All Fields] OR "diagnostical"[All Fields] OR "diagnostically"[All Fields] OR "diagnostics"[All Fields]) AND ("perform"[All Fields] OR "performable"[All Fields] OR "performance"[All Fields] OR "performance s"[All Fields] OR "performances"[All Fields] OR "performative"[All Fields] OR "performatively"[All Fields] OR "performatives"[All Fields] OR "performativities"[All Fields] OR "performativity"[All Fields] OR "performed"[All Fields] OR "performer"[All Fields] OR "performers"[All Fields] OR "performing"[All Fields] OR "performs"[All Fields])) OR (("diagnosis"[MeSH Terms] OR "diagnosis"[All Fields] OR "diagnostic"[All Fields] OR "diagnostical"[All Fields] OR "diagnostically"[All Fields] OR "diagnostics"[All Fields]) AND ("accuracies"[All Fields] OR "accuracy"[All Fields])) OR ("sensitive"[All Fields] OR "sensitively"[All Fields] OR "sensitives"[All Fields] OR "sensitivities"[All Fields] OR "sensitivity and specificity"[MeSH Terms] OR ("sensitivity"[All Fields] AND "specificity"[All Fields]) OR "sensitivity and specificity"[All Fields] OR "sensitivity"[All Fields]) OR ("sensitivity and specificity"[MeSH Terms] OR ("sensitivity"[All Fields] AND "specificity"[All Fields]) OR "sensitivity and specificity"[All Fields] OR "specificity"[All Fields] OR "specific"[All Fields] OR "specifically"[All Fields] OR "specification"[All Fields] OR "specifications"[All Fields] OR "specificities"[All Fields] OR "specifics"[All Fields] OR "specifities"[All Fields] OR "specifity"[All Fields])) AND ("biomarker s"[All Fields] OR "biomarkers"[Supplementary Concept] OR "biomarkers"[All Fields] OR "biomarker"[All Fields] OR "biomarkers"[MeSH Terms])) OR ("mediated"[All Fields] OR "mediational"[All Fields] OR "mediator"[All Fields] OR "mediators"[All Fields] OR "negotiating"[MeSH Terms] OR "negotiating"[All Fields] OR "mediate"[All Fields] OR "mediates"[All Fields] OR "mediating"[All Fields] OR "mediation"[All Fields] OR "mediations"[All Fields])) AND (("perioperative"[All Fields] OR "perioperatively"[All Fields]) AND ("hypersensitiveness"[All Fields] OR "hypersensitivity"[MeSH Terms] OR "hypersensitivity"[All Fields] OR "hypersensitive"[All Fields] OR "hypersensitivities"[All Fields] OR "hypersensitization"[All Fields] OR "hypersensitize"[All Fields])))	102684

	OR (("perioperative"[All Fields] OR "perioperatively"[All Fields]) AND ("anaphylaxis"[MeSH Terms] OR "anaphylaxis"[All Fields])) OR (("perioperative"[All Fields] OR "perioperatively"[All Fields]) AND ("allergie"[All Fields] OR "hypersensitivity"[MeSH Terms] OR "hypersensitivity"[All Fields] OR "allergies"[All Fields] OR "allergy"[All Fields] OR "allergy and immunology"[MeSH Terms] OR ("allergy"[All Fields] AND "immunology"[All Fields]) OR "allergy and immunology"[All Fields])) OR (("anaesthesia"[All Fields] OR "anesthesia"[MeSH Terms] OR "anesthesia"[All Fields] OR "anaesthesias"[All Fields] OR "anesthesias"[All Fields]) AND ("hypersensitiveness"[All Fields] OR "hypersensitivity"[MeSH Terms] OR "hypersensitivity"[All Fields] OR "hypersensitive"[All Fields] OR "hypersensitivities"[All Fields] OR "hypersensitization"[All Fields] OR "hypersensitize"[All Fields])) OR (("anaesthesia"[All Fields] OR "anesthesia"[MeSH Terms] OR "anesthesia"[All Fields] OR "anaesthesias"[All Fields] OR "anesthesias"[All Fields]) AND ("anaphylaxis"[MeSH Terms] OR "anaphylaxis"[All Fields])) OR (("anaesthesia"[All Fields] OR "anesthesia"[MeSH Terms] OR "anesthesia"[All Fields] OR "anaesthesias"[All Fields] OR "anesthesias"[All Fields]) AND ("allergie"[All Fields] OR "hypersensitivity"[MeSH Terms] OR "hypersensitivity"[All Fields] OR "allergies"[All Fields] OR "allergy"[All Fields] OR "allergy and immunology"[MeSH Terms] OR ("allergy"[All Fields] AND "immunology"[All Fields]) OR "allergy and immunology"[All Fields]) AND ("tryptases"[Supplementary Concept] OR "tryptases"[All Fields] OR "tryptase"[All Fields] OR "tryptases"[MeSH Terms])) OR ("histamin"[All Fields] OR "histamine"[Supplementary Concept] OR "histamine"[All Fields] OR "histamine"[MeSH Terms] OR "histamines"[All Fields] OR "histaminic"[All Fields] OR "histaminics"[All Fields])	
#2	(("diagnosis"[MeSH Terms] OR "diagnosis"[All Fields] OR "diagnostic"[All Fields] OR "diagnostical"[All Fields] OR "diagnostically"[All Fields] OR "diagnostics"[All Fields]) AND ("perform"[All Fields] OR "performable"[All Fields] OR "performance"[All Fields] OR "performance s"[All Fields] OR "performances"[All Fields] OR "performative"[All Fields] OR "performatively"[All Fields] OR "performatives"[All Fields] OR "performativities"[All Fields] OR "performativity"[All Fields] OR "performed"[All Fields] OR "performer"[All Fields] OR "performers"[All Fields] OR "performing"[All Fields] OR "performs"[All Fields]) AND ("biomarker s"[All Fields] OR "biomarkers"[Supplementary Concept] OR "biomarkers"[All Fields] OR "biomarker"[All Fields] OR "biomarkers"[MeSH Terms]) AND ("mediated"[All Fields] OR "mediational"[All Fields] OR "mediator"[All Fields] OR "mediators"[All Fields] OR "negotiating"[MeSH Terms] OR "negotiating"[All Fields] OR "mediate"[All Fields] OR "mediates"[All Fields] OR "mediating"[All Fields] OR "mediation"[All Fields] OR "mediations"[All Fields]) AND (("perioperative"[All Fields] OR "perioperatively"[All Fields])	640,828

	AND ("hypersensitiveness"[All Fields] OR "hypersensitivity"[MeSH Terms] OR "hypersensitivity"[All Fields] OR "hypersensitive"[All Fields] OR "hypersensitivities"[All Fields] OR "hypersensitization"[All Fields] OR "hypersensitize"[All Fields]))) OR ("anaphylaxis"[MeSH Terms] OR "anaphylaxis"[All Fields]) OR ("allergie"[All Fields] OR "hypersensitivity"[MeSH Terms] OR "hypersensitivity"[All Fields] OR "allergies"[All Fields] OR "allergy"[All Fields] OR "allergy and immunology"[MeSH Terms] OR ("allergy"[All Fields] AND "immunology"[All Fields]) OR "allergy and immunology"[All Fields])	
#3	("diagnosis"[MeSH Terms] OR "diagnosis"[All Fields] OR "diagnostic"[All Fields] OR "diagnostical"[All Fields] OR "diagnostically"[All Fields] OR "diagnostics"[All Fields]) AND ("perform"[All Fields] OR "performable"[All Fields] OR "performance"[All Fields] OR "performance s"[All Fields] OR "performances"[All Fields] OR "performative"[All Fields] OR "performatively"[All Fields] OR "performatives"[All Fields] OR "performativities"[All Fields] OR "performativity"[All Fields] OR "performed"[All Fields] OR "performer"[All Fields] OR "performers"[All Fields] OR "performing"[All Fields] OR "performs"[All Fields]) AND ("mediated"[All Fields] OR "mediational"[All Fields] OR "mediator"[All Fields] OR "mediators"[All Fields] OR "negotiating"[MeSH Terms] OR "negotiating"[All Fields] OR "mediate"[All Fields] OR "mediates"[All Fields] OR "mediating"[All Fields] OR "mediation"[All Fields] OR "mediations"[All Fields]) AND (("anaesthesia"[All Fields] OR "anesthesia"[MeSH Terms] OR "anesthesia"[All Fields] OR "anaesthesias"[All Fields] OR "anesthesias"[All Fields]) AND ("hypersensitiveness"[All Fields] OR "hypersensitivity"[MeSH Terms] OR "hypersensitivity"[All Fields] OR "hypersensitive"[All Fields] OR "hypersensitivities"[All Fields] OR "hypersensitization"[All Fields] OR "hypersensitize"[All Fields]))	63

WOS- last check 13.03.2026

Table S2: Web of Science

Ordinal Number	Search Strategy	No. of entries
#1	**((((((((((TS = Diagnostic performance) OR (TS = Diagnostic accuracy)) OR (TS = Sensitivity)) OR ((TS = Specificity) AND (TS = Biomarker))) OR ((TS = Mediator) AND (TS = Perioperative hypersensitivity))) OR (TS = Perioperative anaphylaxis)) OR (TS = Perioperative allergy)) OR (TS = anaesthesia hypersensitivity)) OR (TS = anaesthesia anaphylaxis)) OR (TS = anaesthesia allergy tryptase)) OR (TS = Histamine))**	2,476,152
#2	TS=(Diagnostic Anaesthesia hypersensitivity)	130

Link: https://www.cochranelibrary.com/advanced-search/search-manager?search=7952901
Table S3.

Ordinal Number	Search Strategy	Nbr. of entries
#1	MeSH descriptor: [Diagnosis] explode all trees	480018
#2	MeSH descriptor: [Sensitivity and Specificity] explode all trees	22011
#3	"diagnosis":ti,ab,kw OR "diagnostic":ti,ab,kw OR "diagnostical":ti,ab,kw OR "diagnostically":ti,ab,kw OR "diagnostics":ti,ab,kw OR "perform":ti,ab,kw OR "performable":ti,ab,kw OR "performance":ti,ab,kw OR "performance's":ti,ab,kw OR "performances":ti,ab,kw OR "performative":ti,ab,kw OR "performatively":ti,ab,kw OR "performatives":ti,ab,kw OR "performativities":ti,ab,kw OR "performativity":ti,ab,kw OR "performed":ti,ab,kw OR "performer":ti,ab,kw OR "performer's":ti,ab,kw OR "performers":ti,ab,kw OR "performing":ti,ab,kw OR "performs":ti,ab,kw OR "accuracies":ti,ab,kw OR "accuracy":ti,ab,kw OR "sensitive":ti,ab,kw OR "sensitively":ti,ab,kw OR "sensitives":ti,ab,kw OR "sensitivities":ti,ab,kw OR ("sensitivity" AND "specificity") OR "sensitivity and specificity":ti,ab,kw OR "sensitivity":ti,ab,kw OR "specificity":ti,ab,kw OR "specific":ti,ab,kw OR "specifically":ti,ab,kw OR "specification":ti,ab,kw OR "specifications":ti,ab,kw OR "specificities":ti,ab,kw OR "specifics":ti,ab,kw OR "specifities":ti,ab,kw OR "specifity":ti,ab,kw	819399
#4	#1 OR #2 OR #3	1061116
#5	MeSH descriptor: [Biomarkers] explode all trees	32549
#6	MeSH descriptor: [Tryptases] explode all trees	92
#7	MeSH descriptor: [Histamine] explode all trees	1312
#8	"biomarker's":ti,ab,kw OR "biomarkers":ti,ab,kw OR "biomarker":ti,ab,kw OR "mediated":ti,ab,kw OR "mediational":ti,ab,kw OR "mediator":ti,ab,kw OR "mediator's":ti,ab,kw OR "mediators":ti,ab,kw OR "mediate":ti,ab,kw OR "mediates":ti,ab,kw OR "mediating":ti,ab,kw OR "mediation":ti,ab,kw OR "mediations":ti,ab,kw OR "tryptases":ti,ab,kw OR "tryptase":ti,ab,kw OR "histamin":ti,ab,kw OR "histamine":ti,ab,kw OR "histamines":ti,ab,kw OR "histamine's":ti,ab,kw OR "histaminic":ti,ab,kw OR "histaminics":ti,ab,kw	116822
#9	#5 OR #6 OR #7 OR #8	123272

#10	MeSH descriptor: [Anaphylaxis] explode all trees	290
#11	MeSH descriptor: [Hypersensitivity] explode all trees	26800
#12	MeSH descriptor: [Allergy and Immunology] explode all trees	64
#13	"anaphylaxis":ti,ab,kw OR "anaphylactic":ti,ab,kw OR "allergie":ti,ab,kw OR "allergies":ti,ab,kw OR "allergy":ti,ab,kw OR ("allergy" AND "immunology") OR "allergy and immunology":ti,ab,kw OR "hypersensitivity":ti,ab,kw OR "hypersensitiveness":ti,ab,kw OR "hypersensitive":ti,ab,kw OR "hypersensitivities":ti,ab,kw OR "hypersensitization":ti,ab,kw OR "hypersensitize":ti,ab,kw	35986
#14	#10 OR #11 OR #12 OR #13	53654
#15	MeSH descriptor: [Perioperative Period] explode all trees	11599
#16	MeSH descriptor: [Anesthesia] explode all trees	25557
#17	"perioperative":ti,ab,kw OR "peri-operative":ti,ab,kw OR "perioperatively":ti,ab,kw OR "peri-operatively":ti,ab,kw OR "anaesthesia":ti,ab,kw OR "anesthesia":ti,ab,kw OR "anaesthesias":ti,ab,kw OR "anesthesias":ti,ab,kw OR "peroperative":ti,ab,kw OR "peroperatively":ti,ab,kw OR "intraoperative":ti,ab,kw OR "intra-operative":ti,ab,kw OR "intraoperatively":ti,ab,kw OR "intra-operatively":ti,ab,kw OR "intraanestheisa":ti,ab,kw OR "intra-anesthesia":ti,ab,kw OR "intraanaesthesia":ti,ab,kw OR "intra-anaesthesia":ti,ab,kw	152018
#18	#15 OR #16 OR #17	160149
#19	#4 AND #9 AND #14 AND #18	60
