# Supplementary material for: Diagnostic Performance of Biomarkers for Perioperative Hypersensitivity Reactions in Adults: A Systematic Review and Meta-Analysis on Tryptase and Histamine Dosing
Source: Diagnostics (Basel). 2026 Mar 27;16(7):1013. doi: 10.3390/diagnostics16071013 (PMC13073476; doi:10.3390/diagnostics16071013)

Supplementary file S2

Quadas evaluation

Figure S1. Histamine QUADAS plot

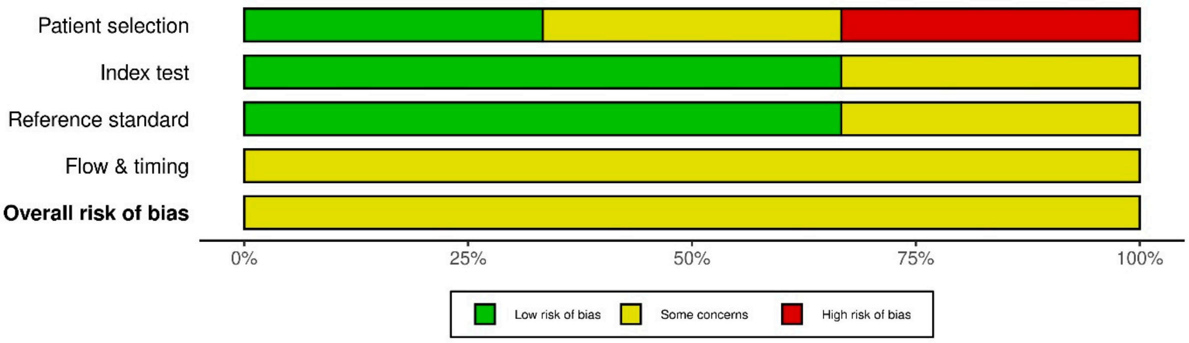

Figure S2. QUADAS traffic light histamine

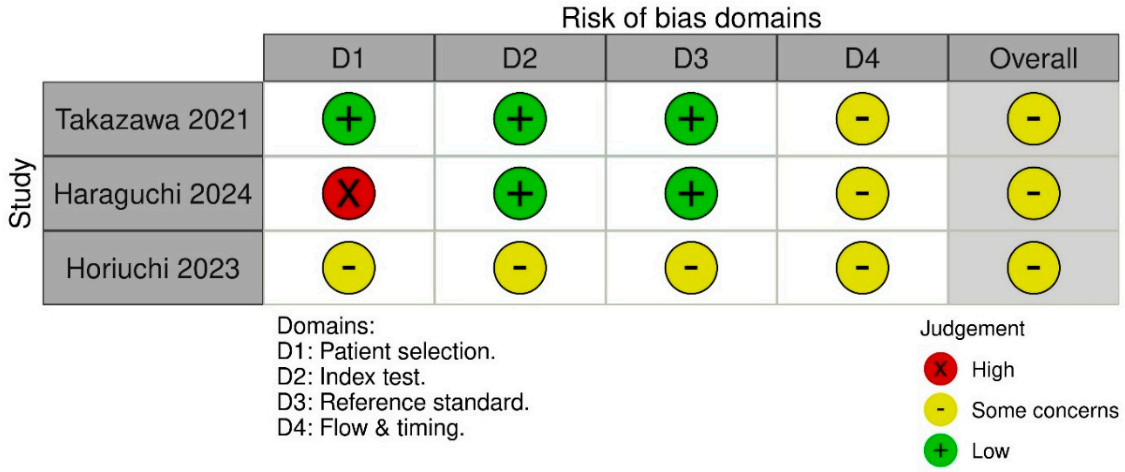

Figure S3. Tryptase plot

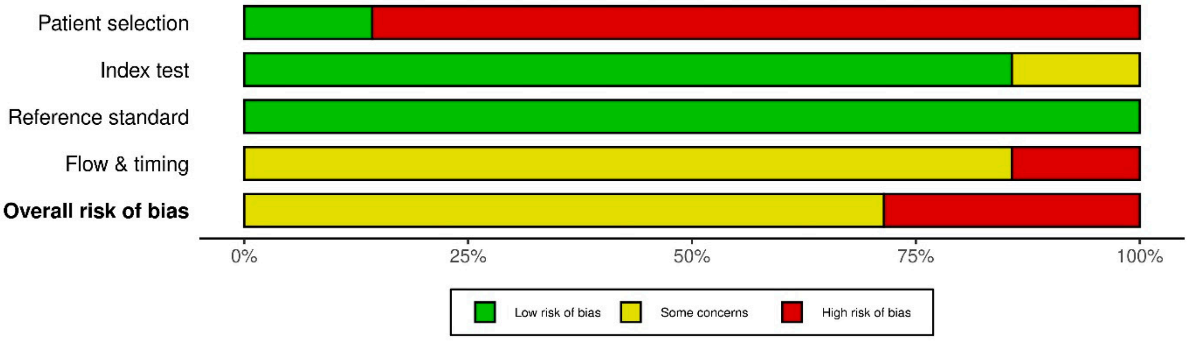

Figure S4. QUADAS risk of bias domains for tryptase

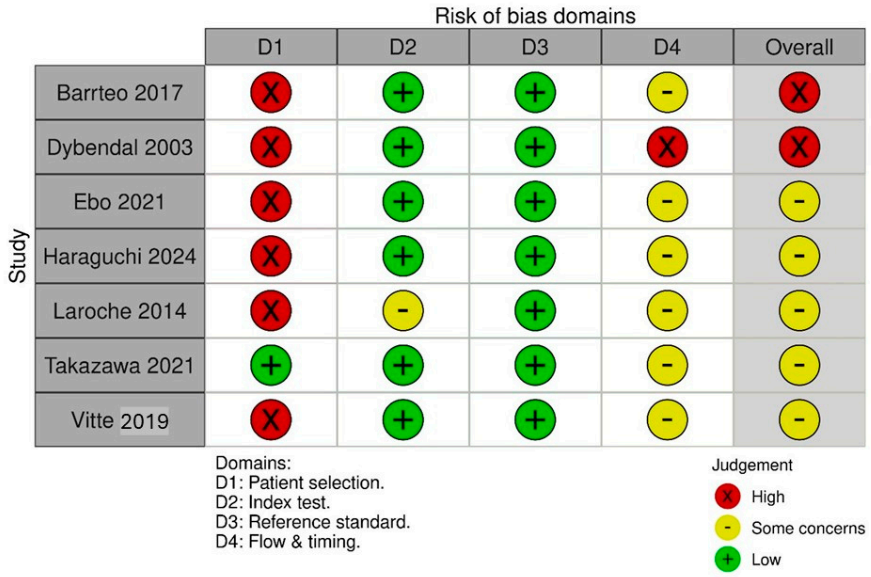

Supplement: Supplementary file 1 [file diagnostics-16-01013-s001.zip › Supplementary File S2.pdf]
